# Supplementary material for: Curated and harmonised transcriptomics datasets of interstitial lung diseases
Source: Data Brief. 2025 Oct 14;63:112139. doi: 10.1016/j.dib.2025.112139 (PMC12581653; doi:10.1016/j.dib.2025.112139)
Supplement: Supplementary file 1 [file mmc1.zip › Supplementary_material/RNA-seq/fastQC reports/GSE138283/TRIMMED_SRR10212995_fastqc.html]

TRIMMED\_SRR10212995.fastq.gz FastQC Report 

FastQC Report

Thu 5 Oct 2023  
TRIMMED\_SRR10212995.fastq.gz

## Summary

- Basic Statistics
- Per base sequence quality
- Per sequence quality scores
- Per base sequence content
- Per sequence GC content
- Per base N content
- Sequence Length Distribution
- Sequence Duplication Levels
- Overrepresented sequences
- Adapter Content

## Basic Statistics

| Measure | Value |
| --- | --- |
| Filename | TRIMMED\_SRR10212995.fastq.gz |
| File type | Conventional base calls |
| Encoding | Sanger / Illumina 1.9 |
| Total Sequences | 82588412 |
| Sequences flagged as poor quality | 0 |
| Sequence length | 60-76 |
| %GC | 46 |

## Per base sequence quality

## Per sequence quality scores

## Per base sequence content

## Per sequence GC content

## Per base N content

## Sequence Length Distribution

## Sequence Duplication Levels

## Overrepresented sequences

| Sequence | Count | Percentage | Possible Source |
| --- | --- | --- | --- |
| CCACAAATTATGCAGTCGAGTTTCCCACATTTGGGGAAATCGCAGGGGTCAGCACATCCGGAGTGCAATGGATA | 622816 | 0.7541203238052331 | No Hit |
| CCCCACTACCACAAATTATGCAGTCGAGTTTCCCACATTTGGGGAAATCGCAGGGGTCAGCACATCCGGAGTGCA | 465563 | 0.5637146770663177 | No Hit |
| CCCACTACCACAAATTATGCAGTCGAGTTTCCCACATTTGGGGAAATCGC | 350650 | 0.42457530240441 | No Hit |
| CACAAATTATGCAGTCGAGTTTCCCACATTTGGGGAAATCGCAGGGGTCA | 297222 | 0.3598834180272167 | No Hit |
| CTCAGACCGCGTTCTCTCCCTCTCACTCCCCAATACGGAGAGAAGAACGATCATCAATGGCTGACGGCAGTTGC | 241659 | 0.29260642522101044 | No Hit |
| CTCCGTTTCCGACCTGGGCCGGTTCACCCCTCCTTAGGCAACCTGGTGGT | 224775 | 0.272162879218455 | No Hit |
| CCTTAGGCAACCTGGTGGTCCCCCGCTCCCGGGAGGTCACCATATTGATG | 208306 | 0.25222182501826046 | No Hit |
| CCCCTCCTTAGGCAACCTGGTGGTCCCCCGCTCCCGGGAGGTCACCATAT | 184384 | 0.22325650237711314 | No Hit |
| CTGGAGTCTTGGAAGCTTGACTACCCTACGTTCTCCTACAAATGGACCTTGAGAGCTTGTTTGGAGGTTCTAGC | 147321 | 0.1783797465436192 | No Hit |
| CCAGGCTGGAGTGCAGTGGCTATTCACAGGCGCGATCCCACTACTGATCA | 139968 | 0.16947656046468118 | No Hit |
| GTCGAGTTTCCCACATTTGGGGAAATCGCAGGGGTCAGCACATCCGGAGTGCAATGGATAAGCCTCGCCCTGGGA | 135760 | 0.1643814146711042 | No Hit |
| CCCTCCTTAGGCAACCTGGTGGTCCCCCGCTCCCGGGAGGTCACCATATT | 133527 | 0.16167764552731684 | No Hit |
| CTACCACAAATTATGCAGTCGAGTTTCCCACATTTGGGGAAATCGCAGGG | 109595 | 0.13270021464996806 | No Hit |
| CTCAGGCTGGAGTGCAGTGGCTATTCACAGGCGCGATCCCACTACTGATC | 109430 | 0.1325004287526439 | No Hit |
| CTCCTTAGGCAACCTGGTGGTCCCCCGCTCCCGGGAGGTCACCATATTGA | 103235 | 0.12499937642583563 | No Hit |
| CCTCCTTAGGCAACCTGGTGGTCCCCCGCTCCCGGGAGGTCACCATATTGATGCCGAACTTAGTGCGGACACCCG | 102512 | 0.12412395094846963 | No Hit |
| GTCCCCCACTACCACAAATTATGCAGTCGAGTTTCCCACATTTGGGGAAA | 90026 | 0.10900560722731902 | No Hit |
| CACTACCACAAATTATGCAGTCGAGTTTCCCACATTTGGGGAAATCGCAG | 88501 | 0.10715910120659544 | No Hit |
| CCACTACCACAAATTATGCAGTCGAGTTTCCCACATTTGGGGAAATCGCA | 87069 | 0.10542520178254547 | No Hit |
| CCCCCACTACCACAAATTATGCAGTCGAGTTTCCCACATTTGGGGAAATCGCAGGGGTCAGCACATCCGGAGTGC | 86068 | 0.10421316733877871 | No Hit |

## Adapter Content

Produced by FastQC (version 0.11.7)
